# Case report: Musculoskeletal metastastic inflammatory myofibroblastic tumor (IMT) treated by sequential ALK-TKI with longterm response

**DOI:** 10.3389/fonc.2024.1505257

**Published:** 2025-01-27

**Authors:** Thomas Cochin, Sabine Noal, Dinu Stefan, Damien Bodet, Jérémie Rouger, Marine Dorbeau, Zoé Neviere

**Affiliations:** ^1^ Department of Radiation Oncology, Centre François Baclesse, Caen, France; ^2^ Department of Sarcoma Oncology, Centre François Baclesse, Caen, France; ^3^ Department of Pediatry Oncology, Centre Hospitalier Universitaire, Caen, France; ^4^ Department of Pathology, Centre François Baclesse, Caen, France

**Keywords:** inflammatory myofibroblastic tumor, crizotinib, sequential treatment ALK-TKI, pediatric, ALK inhibitor

## Abstract

Inflammatory myofibroblastic tumors (IMTs) are known to be associated with rearrangements of the anaplastic lymphoma kinase (ALK) gene. The treatment of this type of tumor includes systemic therapies such as chemotherapies or anti-inflammatories; in recent years, targeted anti-ALK therapies have emerged and became the standard of care in ALK rearranged patients. We aimed to present a rare case of musculoskeletal IMT with ALK rearrangement, characterized by metastatic evolution and enhanced responses to sequential treatment with all ALK-TKI. We have outlined a potential treatment pathway involving sequential ALK-TKI targeted therapies and successive local interventions to control the cancer.

## 1 Introduction

Inflammatory myofibroblastic tumors (IMT) represent an histologic subtype of sarcoma consisting mainly of myofibroblastic proliferation along with varying amount plasma and lymphocyte inflammatory cells (1, 2). Amplification of the ALK receptor, located on chromosome 2p23, is observed in 55-66% of IMT (2, 3). Others targetable tyrosine kinase in the same pathway, such as ROS or NTRAK are also identified (4, 5). Formerly classified as benign tumors, they are now classified as intermediate malignant potential. They can exhibit locoregional and metastatic aggressiveness, particularly in case of extrapulmonary lesions (between 5% and 25%) (6, 7).

IMT preferentially affect lungs, mesentery and orbits but they have been reported in various sites. Musculoskeletal locations are rarely described. They predominantly affect children and young adults, with a rather balanced sex ratio (3, 8, 9).The diagnosis is histological by excisional biopsy.

Therapeutic recommendations for IMT are controversial and encompass various modalities, including surgery, radiotherapy, chemotherapy, immune-modulators/immune-suppressors, and more recently, Tyrosine Kinase Inhibitors (TKI) targeting the ALK receptor (TKI-ALK) (2).

To the best of our knowledge, few cases reported the use of second or third generation ALK-TKI and describe therapeutic sequence of these treatment in IMT (10, 11).

## 2 Case report

**Figure 1:**
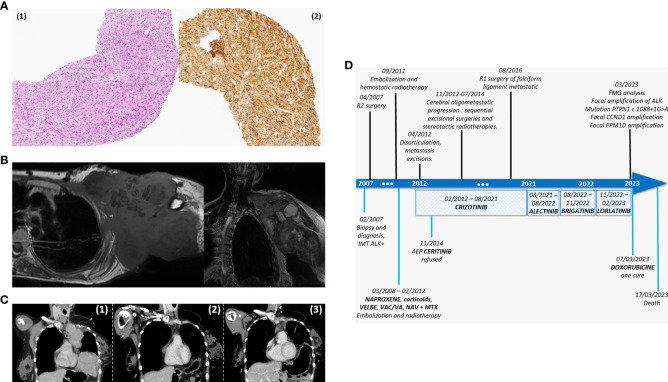
**(A)** IMT, digital pictures of hematoxylin and eosin (1) and immunohistochemistry with ALK showing strong cytoplasmic staining x20 magnification (2). **(B)** MRI in 01/2012 before the disarticulation surgery, axial and coronal T1. **(C)** Major partial response at treatment by ALECTINIB, coronal CT-scan 08/2021 (1) versus 11/2021 (2) and 11/05/2022 (3). **(D)** Chronologic evolution of treatments among our patient. In the upper part are the surgeries and different radiotherapies have been performed, in the lower part are the diagnosis and different chemotherapies/anti-ALK oral therapies.

In February 2007, a 15-years-old female patient presented inflammatory symptoms and pain on axillary and proximal left humeral region. MRI revealed an 8-centimeter lesion in the axilla and one-third proximal to the humerus with close proximity to the axillary nerves and vessels. PET-scanner did not identify any other suspicious lesions. A micro-biopsy was performed, which concluded to a fibromatous tumor (**Figure 1A**). A second read in expert networks RRePS specified the diagnostic of IMT which high ALK immunohistochemistry expression.

The patient underwent an uncompleted R2 surgery in April 2007 due to proximity of vessels (**Figure 1B**). She experienced a relapse in Mars 2008 and was treated with 3 months of anti-inflammatory (NAPROXENE) and 1 month of corticosteroids. Five months later, radiological progression led to proposal of a six months regimen of alternative endoxan/vincristine/actinomycine and vincristine/actinomycine, which allowed for tumor stabilization. After six months of monitoring, local progression was observed and the patient received 40 cycles of navelbine/methotrexate. In September 2011, the patient presented a major hemorrhage. An embolization of six axillaries vessels was followed by a conventional hemostatic radiotherapy with 54 Gy administered over 25 sessions.

In February 2012, a local progression and new hepatic, left adrenal gland, and two cerebral lesions were been observed. CRIZOTINIB treatment was initiated, leading to tumor stabilization. Successive surgeries were performed, including left-arm disarticulation, excision of left adrenal gland, and removal of hepatic metastasis in April and July 2013. Histologic examination revealed a Ki67 at 30% in the disarticulation piece, histologically confirming of malignant IMT. Finally, no tumor was found in the left adrenal gland.

Cerebral oligometastatic progression led to sequential excisional surgeries and stereotactic body radiation therapies (SBRT) in 2012 until 2014.

As a result of pursuing treatment for oligoprogression, a request was made to access to CERITINIB (which was studied in Non-Small Cell Lung Cancer (NSCLC) with ALK rearrangement) through an expanded access program in 2014, but was refused in November (12). Ultimately, the patient underwent frontal SBRT, CRIZOTINIB treatment has been continued.

During this time on CRIZOTINIB, the patient experienced several adverse events, including grade IV neutropenia, grade II hepatic cytolysis, and grade II nutritional toxicities which led to a dose adjustment of CRIZOTINIB, with durable acceptable tolerability thereafter.

In August 2016, a CT scan revealed the appearance of falciform ligament metastasis, leading to an R1 surgery and the pursuing of CRIZOTINIB until August 2021.

Subsequently, rapid pleuro-pulmonary progression had been noted on the CT scan, translating clinically by worsening of the general state and nights sweats, prompting the proposal of a second-generation ALK-TKI, ALECTINIB. This treatment resulted in a significant partial radiologic response and clinical improvement, after three months of treatment, without significant toxicity (**Figure 1C**). However, one year later, focal progression of para-cardiac lung nodule was observed. Initially, cryotherapy was planned but contraindicated due to the growth kinetics, leading to the consideration of a third-line ALK-TKI, BRIGATINIB. Unfortunately, after three months of this treatment, BRIGATINIB induced several toxicities (grade I cytolysis, grade II diarrhea and asthenia) and did not demonstrate any efficacy.

In September 2022, a biomolecular analysis was initiated to search for resistance to ALK inhibitors (*EGFR*, *MET*, *KRAS*, *BRAF*, *NRAS*, *HER2* (*ERBB2*) and *PIK3CA*) but yielded inconclusive results.

LORLATINIB treatment was started in January 2023, despite the absence of toxicity, it was been ineffective after one month. Chemotherapy with DOXORUBICIN was been administered promptly in March 2023.

Simultaneously, whole genome analysis was conducted, revealing a focal *ALK* amplification, a mutation on *PTPN1* c.1088 + 1G>A, a focal *CCND1* amplification, and a focal *PPM1D* amplification.

Unfortunately, the patient succumbed to a septic shock, ten days after the first DOXORUBICIN cycle, marking sixteen years since her initial diagnosis and nine years since the onset of metastases. Evolution of these different treatments is represented in **Figure 1D**.

## 3 Discussion

It’s the first report of a long-term response to sequential ALK-TKI treatment in metastatic IMT.

As a reminder, the typical presentation of inflammatory myofibroblastic tumors is: locoregional progression with possible metastases, primarily in the lung or abdominopelvic region, affecting children and young adults, with no gender predilection, and with ALK receptor amplification observed in half of all cases.

This patient presented an original and unusual manifestation of IMT, previously described by Gaudichon et al. in 2016 (13).

Indeed, the patient was a young adult woman with axillary musculoskeletal lesion and metastatic progression. ALK rearrangement was identified from the onset of the tumor’s history by immunochemistry and confirmed later on molecular genomic analysis.

Due to the similar presence of ALK rearrangements in IMT and NSCLC, the rational of ALK-targeted treatment can be anticipated (10).

Butrynski et al. reported in 2010 the first effective response in the case of a CRIZOTINIB-treated IMT patient who remained without progression for eight months according to RECIST criteria (14).

Then, two phase II studies have confirmed the efficacy of CRIZOTINIB in locally advanced or metastatic IMT among pediatric population: Mosse et al. and Schöffski et al. with an update at 3 years (15–17). The first study included ALK-positive IMT and showed an overall response rate of 86% and a complete response rate of 36% (15). The second study reported an objective response rate of 66.7% in ALK-positive patients (17).

Therefore, CRIZOTINIB has become the standard of care in advanced ALK-rearranged IMT (10).

In regards to the state of knowledge concerning the ALK-TKI in IMT, our patient benefited early of these new therapies, in February 2012. Interestingly, the patient grew alongside the evolution of generations of targeted ALK-TKI therapies.

The interesting aspect of this case was also the protracted duration (9.6 years or 115 months) of CRIZOTINIB treatment of our patient. This duration exceeds those reported in the literature: in the trial by Mosse et al., the median duration was 1.63 years with a standard deviation of 0.55 to 2.30 years, and the longest reported response was 5.25 years or 63 months (15). In the trial by Schöffski et al., update in 2021, the median duration was 0.58 years in ALK-positive patient with a standard deviation of 0.25 to 1.42 years, and the longest reported responses on CRIZOTINIB exceeding five years or 60 months (16, 17).

Furthermore, in our patient, the partial response to CRIZOTINIB allowed multiples local treatments for oligometastatic brain progression over a period of seven years before switching systemic treatment (from July 2014 to August 2021), while maintaining a good performance status and quality of life.

This type of treatment sequence has been described in ALK rearrangement NSCLC treated by ALK-TKI. It involves two aspects: the first one is systemic therapy such as targeted ALK-TKI, and the second one is local ablative therapy, such as SBRT, radiosurgery, surgical resection or whole brain radiation therapy (WBRT), which allows for the continuation of the same systemic therapy (18).

It should be noted that the efficacy of CRIZOTINIB in brain metastases was lower than in other body sites. In NSCLC, the central nervous system was often the first site of oligoprogression on CRIZOTINIB treatment, with partial response in other lesions (19). Consequently, combining CRIZOTINIB with local ablative treatment seems quite attractive.

Some authors even advocate for a “combined model of ALK-TKI with radiotherapy”, particularly in the case of brain metastases, using techniques such as SBRT or WBRT. This approach allows the continuation of the same ALK-TKI therapy while providing additional time to control the disease (19, 20).

This approach may prove to be an interesting treatment strategy in IMT with ALK rearrangement, similar to strategies employed in NSCLC brain progression.

Also, note that the patient in the first case report by Butrynski et al. presented disease progression after eight months on CRIZOTINIB with three abdominal masses. The team performed a surgical resection followed by restarting CRIZOTINIB, resulting in a long-term complete response (8, 14).

Beyond the first-line CRIZOTINIB, there is limited information on other lines of ALK inhibitors among IMT patients.

In the review by Wang et al. in 2021, three case reports have demonstrated the efficacy of ALECTINIB or LORLATINIB in IMT (9).

None have experimented BRIGATINIB (To note: a clinical trial, NCT04925609, is currently in progress to study the efficacy of BRIGATINIB in a population of under 25-year-olds with ALK rearrangement with lymphoma, IMT or others solid tumors).

Regarding the efficacy and therapeutic sequence of ALK inhibitors among IMT patients, the use of ALECTINIB follows the same rational as in treatment of NSCLC after progression on CRIZOTINIB (10, 21).

Yuan et al. described a similar situation in 2017 with sequential treatment of ALK inhibitors in a patient with brain metastatic IMT at diagnosis. They used all three generation of ALK-TKI with CRIZOTINIB, CERITINIB, ALECTINIB and LORLATINIB resulting in a complete response (11).

Wang et al. are proposing different strategies for sequential treatments in IMT, whether to start with CRIZOTINIB or not, but there is a lack of evidence to recommended a specific therapeutic sequence for the time being (10).

The adverse effects presented by our patient under the different generations of TKI were comparable to those found in the IMT or NSCLC trials (neutropenia, diarrhea, nausea, asthenia, etc.) (15, 17, 21).

Lastly, in this case, BRIGATINIB and LORLATINIB did not shown efficacy. This could be explained by a specific mutation of *PTPN1* identified through extended genetic analysis. This alteration has been reported to confer resistance to CRIZOTINIB and second generation ALK inhibitors, leading to resistance through phosphorylation or other activation signaling pathways described in leukemia (22–24). These mechanisms of resistance to ALK inhibitors are also described in NSLSC (25).

To our knowledge, this is the first case of IMT to benefit from sequential treatment with all generations of ALK-TKI, resulting in a long partial response to CRIZOTINIB that enabled successive local interventions on metastases over the years.

## 4 Conclusion

In summary, we presented a rare case of long-responder musculoskeletal inflammatory myofibroblastic tumor with ALK rearrangement and metastatic evolution. This case allowed us to observe an extraordinary partial response lasting nine and a half years on CRIZOTINIB and sequential treatment with all generations of ALK-TKI. In the future, our report suggests that sequential systemic medical treatment with ALK-TKI, combined with successive local treatments, could be considered in IMT patients with oligometastatic progression.

## Data Availability

The original contributions presented in the study are included in the article/supplementary material. Further inquiries can be directed to the corresponding authors.
